# Surface Acoustic Wave Devices for Harsh Environment Wireless Sensing

**DOI:** 10.3390/s130606910

**Published:** 2013-05-24

**Authors:** David W. Greve, Tao-Lun Chin, Peng Zheng, Paul Ohodnicki, John Baltrus, Irving J. Oppenheim

**Affiliations:** 1 National Energy Technology Laboratory, Pittsburgh, PA 15236, USA; E-Mails: tchin@andrew.cmu.edu (T.-L.C); pzheng08@gmail.com (P.Z.), paul.ohodnicki@netl.doe.gov (P.O.); john.baltrus@netl.doe.gov (J.B.); ijo@andrew.cmu.edu (I.J.O.); 2 Department of Electrical and Computer Engineering, Carnegie Mellon University, Pittsburgh, PA 15213, USA; 3 Department of Civil and Environmental Engineering, Carnegie Mellon University, Pittsburgh, PA 15213, USA

**Keywords:** surface acoustic wave, gas sensor, oxygen, langasite, zinc oxide, tin oxide

## Abstract

Langasite surface acoustic wave devices can be used to implement harsh-environment wireless sensing of gas concentration and temperature. This paper reviews prior work on the development of langasite surface acoustic wave devices, followed by a report of recent progress toward the implementation of oxygen gas sensors. Resistive metal oxide films can be used as the oxygen sensing film, although development of an adherent barrier layer will be necessary with the sensing layers studied here to prevent interaction with the langasite substrate. Experimental results are presented for the performance of a langasite surface acoustic wave oxygen sensor with tin oxide sensing layer, and these experimental results are correlated with direct measurements of the sensing layer resistivity.

## 1. Introduction

**Figure 1.**
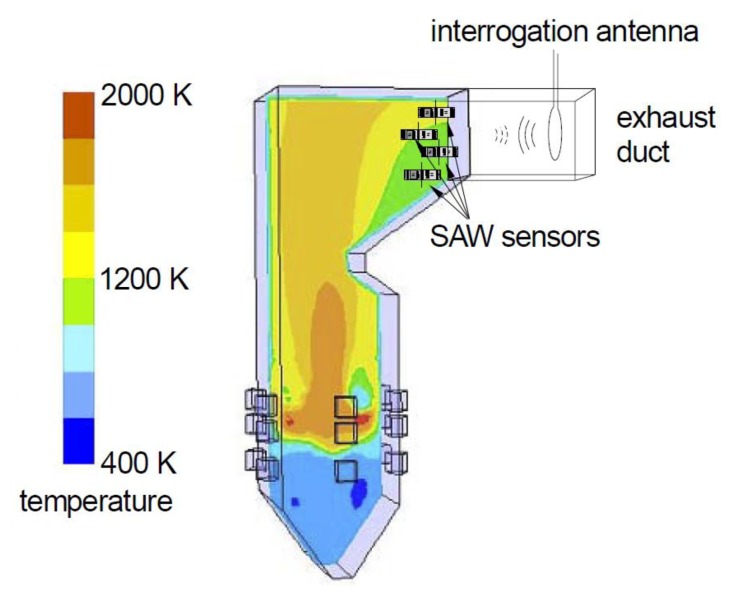
Concept for sensors in the exhaust of an oxy-fuel combustion system (120 MWh, wet recycle). Multiple sensors can be interrogated using a single antenna. Combustion takes place near the bottom with the exhaust at the top and to the right. Colors indicate temperature. Figure adapted from [2].

Harsh environment sensing has received increased attention in recent years. Harsh environment applications require sensors that can survive high temperature, high vibration, and chemically aggressive environments. Often wireless operation is desirable because it simplifies installation by eliminating wiring and penetrations into the harsh environment. This paper addresses the development of temperature and gas sensors for combusion exhaust environments. Potential applications include turbine exhaust [1] and the exhaust of oxy-fuel coal combustors (Figure 1). We will briefly describe the motivation for the second of these applications.

In oxy-fuel combusion, separated oxygen is injected into the combustion environment [3]. Oxy-fuel combustion can result in an exhaust which consists only of carbon dioxide and water vapor. The water vapor is readily condensed and the carbon dioxide can then be sequestered, limiting the release of carbon dioxide into the atmosphere. Carbon dioxide is often stored in oil wells, yielding a secondary benefit of enhanced recovery of the remaining oil. It is important to accurately measure the (uncombusted) oxygen concentration in the exhaust stream so as to minimize the amount of expensive separated oxygen that is consumed. Temperatures in the exhaust can reach 1,000 °C, and it is desirable to place multiple sensors to monitor the oxygen concentration across a large duct with nonuniform temperature and gas concentration.

In principle refractory semiconductors such as aluminum gallium nitride, silicon carbide, or diamond could be used to build sensors and the associated electronics. This may well be possible in the near term for operation at moderately high temperatures (up to 350 °C or so). However reaching significantly above that temperature will be extremely challenging, requiring considerable research into device design, sensor concepts, and especially metallizations and insulators that can survive high temperatures. A final remaining challenge is providing power for active electronics.

Another possibility is the use of passive, inductively coupled sensors [4]. The major problem with this approach for wireless sensing is the limited allowable distance between the sensor and the probe coil, typically limited to the order of the diameter of the coils.

We are led, therefore, to surface acoustic wave sensors. A detailed summary of previous work on surface acoustic wave sensors is presented in a following section. Briefly, surface acoustic wave devices have been demonstrated to operate at elevated temperatures. Gas sensing can be accomplished either by sensing changes in the conductivity of a sensing layer or by observing mass changes when specific molecules are absorbed. Wireless operation can be achieved by sensing varying RF absorption or by using the SAW device in a transponder mode. Finally, frequency diversity or coding can be used to probe multiple sensors with a single antenna. Thus surface acoustic wave sensors can address all of the requirements for harsh-environment applications.

In this paper we will begin by describing the operation of surface acoustic wave devices with particular emphasis on sensing. We will then summarize relevant work on the application of langasite as a substrate material for SAW sensors. We will report our recent work on the development of oxygen gas and temperature sensors using langasite substrates. Finally, we outline some remaining barriers to the development of harsh-environment SAW sensors and the prospects for further development.

## 2. Wired and Wireless Surface Acoustic Wave Transducers

**Figure 2.**
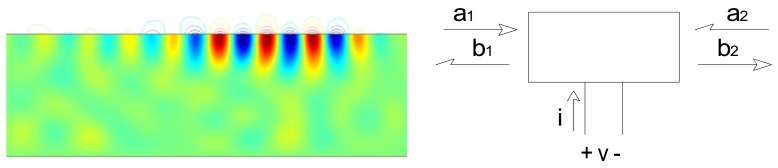
Surface waves: (**Left**) surface wave propagating along the surface (lithium niobate substrate; color = *y* displacement (red, positive, green negative), contour = electric field, computed at a center frequency of 450 MHz); and (**Right**) model of an interdigitated transducer showing incident and reflected waves.

Surface acoustic wave devices consist of one or more interdigitated transducers (IDTs) fabricated on a piezoelectric substrate. The interdigitated transducers consist of interleaved metal fingers that can be excited by an external RF source. Surface acoustic waves can be launched on the piezoelectric substrate by exciting an interdigitated transducer with a sinusoid or a windowed sinusoid (Figure 2). Surface waves have displacements that decay exponentially into the substrate [5].

Upon reaching a second open-circuited interdigitated transducer, some of the ultrasonic wave is reflected back to the emitting transducer. This ultrasonic wave is then converted into an electrical signal. A reflected wave can be detected either in a time-resolved measurement (if a pulse excitation is used) or as its effect on the measured admittance at the emitting IDT terminals (if a steady-state measurement). In either case the round-trip delay time can be determined.

**Figure 3.**
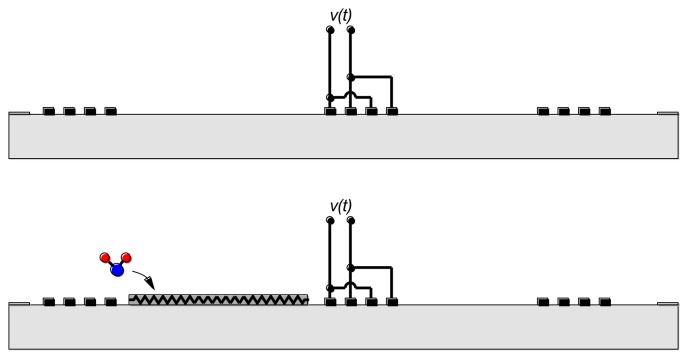
SAW sensors: for sensing of temperature only (**Top**) and for simultaneous gas and temperature sensing (**Bottom**).

The combination of emitting and reflecting SAW IDTs can be used as a transducer when there is a dependence of surface wave velocity on the quantity of interest. Except for a narrow range of temperatures and carefully chosen propagation directions, the surface wave velocity is temperature-dependent and the SAW device can be used as a temperature sensor [6,7] (Figure 3(Top)). Strain also influences the round-trip delay time and has been used to implement strain and pressure sensors [6,7].

Overlayers deposited on the propagation path provide a means for chemical or biological sensing [6,7] (Figure 3(Bottom)). Some overlayers selectively absorb certain chemical or biological species, resulting in a mass change that changes the surface wave velocity. Alternately, propagation of the surface wave results in charges on the surface of the piezoelectric material and these charges produce an electric field protruding from the surface. The interaction between this electric field and the sensing layer influences the surface wave velocity. It is easy to understand the influence of the sensing layer in two limits. When the conductivity of the sensing layer is high, the electric field is shorted by charges induced in the sensing layer. On the other hand, when the sensing layer has low conductivity the electric field causes *piezoelectric stiffening*, resulting in an increase in velocity relative to the velocity with the electric field shorted. For intermediate values of conductivity there is an intermediate change in velocity.

**Figure 4.**
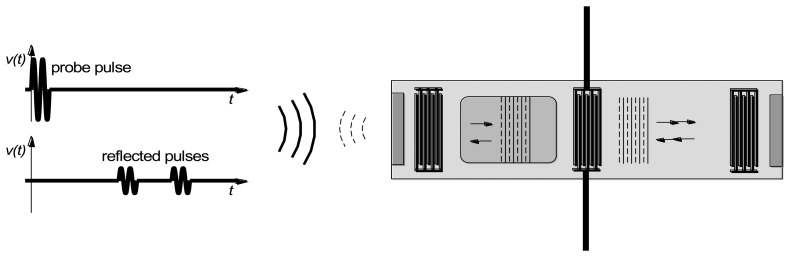
The wireless sensing concept.

As noted earlier, sensing is accomplished by measuring velocity changes either by changes in the terminal admittance (if a steady state measurement), or changes in the round-trip time for pulse propagation. Alternatively it is possible to measure changes in resonant frequency when a two-port device is used as a resonator. In the first two cases sensing can also be done in wireless mode, by replacing the cable between the emitting IDT and the electronics by a wireless link (Figure 4). The SAW device thus acts as a passive transponder, incurring the propagation loss in the RF link twice. As a result signal strength and the resolution of the interrogation electronics is important in obtaining good results.

SAW devices have been made using a wide variety of piezoelectric substrates. In our intended application of harsh environment sensing a substrate material that can tolerate high temperatures is essential. We will focus on langasite, which has been used by a number of recent researchers and which has been used for the experiments to be reported later. In the following, we will first survey relevant aspects of prior work on langasite SAW devices. We will then present some of our recent results on temperature and gas sensing using langasite SAW devices.

## 3. Langasite as a SAW Substrate

Langasite (La_3_Ga_5_SiO_14_) is one of a number of piezoelectric materials [8] recently investigated for high-temperature applications. Langasite has a melting temperature of 1,490 °C and is grown as a single crystal from the melt [8]. The first report on langasite noted that it could have zero temperature coefficient of the elastic vibration frequency near room temperature [9]. Hornsteiner *et al.* remarked on the advantages of langasite for high temperature SAW devices and reported a delay line operating up to 1,000 °C [10]. In a subsequent paper [11] they remarked on the increasing SAW attenuation with temperature and discussed the potential application as an ID tag (wireless mode). A1N [12] and other materials related to langasite [13,14] have also been considered for high-temperature SAW devices although it appears that much of the recent work directed at high-temperature sensing has used langasite.

Particular concerns in the application of langasite for high-temperature sensing include metallization reliability and increased acoustic losses at high temperature. Hornsteiner *et al.* used platinum metallization with a titanium adhesion layer [11]. This metallization is somewhat tolerant of high temperatures and was used to operate a SAW device for short times at 1,000 °C. It has been reported that Ti migrates into Pt when annealed at high temperature [15] in contrast to the behavior of Zr adhesion layers. Thiele *et al.* [16] used Pt and Pd metallization with a Zr adhesion layer. Pt metallization was able to survive 3 weeks at 750 °C without significant degradation. Aubert [17] compared iridium and tantalum as adhesion layers, with better results obtained with tantalum. Somewhat inferior results were later reported for Pt/Ta metallization relative to an Ir/Ti metallization [18]. Da Cunha *et al.* evaluated a Pt/Rh alloy on ZrO_2_ buffer layer, finding improved stability over Pt/Zr metallization [19,20]. They also used an SiAlON passivation layer for improved protection of the electrodes. Long-term stability of langasite SAW resonators with the ZrO_2_/Pt/Rh metallization and SiAlON passivation has been studied for several months at 800 °C and compared with Pt metallization [20]. The platinum metallization showed inferior durability. Generally failure of platinum metallization is due to agglomeration of platinum leading to loss of electrical continuity [19,21]. Aubert *et al.* [21] showed that iridium with a titanium adhesion layer could survive temperatures of at least 1,140 °C, with eventual failure attributed to loss of gallium and oxygen rather than metallization failure. However these experiments were performed in vacuum as iridium is readily oxidized. Thus there are several metallization options applicable above 750 °C, with some metallizations that are usable well above this temperature.

Surface transverse (SH) waves have been studied in langasite but mostly for resonator and liquid sensing applications [22–24]. We will focus on Rayleigh (ordinary) surface waves. The surface wave velocity, temperature coefficient, electromechanical coupling coefficient, and power flow angle vary with the direction of propagation. The propagation direction is usually specified using the Euler angles (*φ*, *θ*, *ψ*). Takeguchi *et al.* [25] reported that the (0°, 140°, 22°–24°) cut had zero temperature coefficent, zero power flow angle, and good electromechanical efficiency. In addition this direction yields a natural single phase unidirectional transducer. Based on experimental and theoretical investigations, Naumenko and coworkers [26,27] recommended the orientation (0°, 138°, 27°) when also considering the anisotropy parameter, which determines the amount of diffraction. This particular cut has been used in much of the SAW sensor work to date. While the temperature coefficient is zero at room temperature, the velocity decreases monotonically above room temperature, making this orientation appropriate for temperature sensing.

While early work [28] was motivated by reduced losses in langasite near room temperature relative to quartz, increasing losses are observed in langasite at higher temperatures. These losses lead to attenuation and are a significant problem in the development of high-temperature sensors. Seh *et al.* [29] measured the quality factor of bulk resonators made from polycrystalline langasite up to 1,000 °C and correlated the observations with electrical conductivity measurements made as a function of oxygen partial pressure. They found decreasing *Q* as the temperature was increased. Extensive measurements also showed that langasite is an oxygen defect conductor with strongly increasing electron conductivity when the oxygen partial pressure is very low. Measurements on single crystal bulk resonators were made by Johnson *et al.* [30] who observed increasing acoustic loss up to 700 K. Schreuer *et al.* [31] also saw increasing loss with temperature in single-crystal langasite and examined the effect of frequency and annealing ambient. An increase in surface wave attenuation in SAW devices with temperature and frequency was subsequently observed [32–34]. Shrena *et al.* reported lower losses for the (0°, 138°, 27°) orientation than the (0°, 30°, 27°) and also higher losses when metal was on the propagation path [33,34]. These acoustic losses represent a significant obstacle to the development of high-temperature sensors as they reduce signal strength at high temperature and limit the maximum frequency of operation.

## 4. Langasite SAW Sensors

Early studies of temperature and gas sensing were performed by da Cuhna and coworkers. They measured the frequency shift of two-port 168 MHz resonators with (0°, 138°, 27°) orientation langasite as a function of temperature up to 750 °C [35]. Readily measurable changes in resonant frequency were observed consistent with theory. A similar device structure was used for gas detection experiments [36]. Resonators with the propagation path covered with platinum showed a positive frequency shift when exposed to C_2_H_4_ at 250–450 °C after pre-oxidation of the Pt surface. This direction of frequency change was consistent with the removal of oxygen from the sensing layer, so the mechanism was sensing of a mass change. A WO_3_ layer over platinum resulted in an increase in sensitivity above that obtained with Pt alone. A similar experiment with a palladium sensing layer showed a reproducible negative frequency change, consistent with a mass change due to absorption of hydrogen. Additional details about these sensors were reported in [37] and showed drift and other complex behavior. The (0°, 138°, 27°) orientation used by da Cuhna *et al.* and many other researchers shows significant temperature dependence. In [38], Thiele *et al.* used two resonators, one exposed to the sensing gas and the other not, to obtain temperature compensation for hydrogen detection at 250 °C. The initial response to hydrogen was rapid but steady state was not reached for 25–75 min. The temperature dependence of resonator frequency for the langasite (0°, 140°, 22–24°) orientation was also studied by Buff *et al.* [39] up to about 600 °C and compared with gallium phosphate SAW devices. Gas detection was also studied by Tortissier *et al.* [40]. They used a delay line with TiO_2_ deposited on the propagation path using an ink-jet printer. They observed drying of the TiO_2_ sensing layer and operated up to 500 °C using an integrated heater.

As noted earlier, a unique advantage of SAW gas and temperature sensors is the ability to operate in wireless mode. Wang *et al.* demonstrated temperature sensing up to 700 °C using a device with an emitting IDT and multiple reflectors [41]. A Pt wire helical dipole antenna was matched to the SAW device using a parallel inductor and operation was achieved with up to 1 m separation. Faschberger *et al.* [42] demonstrated a 2.45 GHz wireless temperature sensor with a slot antenna operated up to 450 °C. Their SAW device had multiple reflectors making it possible to assign codes for sensor identification. Measurements were performed with a custom-built transponder that operated in frequency-swept (CW) mode. Using the phase in addition to magnitude information yielded an improvement in resolution. Measurements with an accuracy of ±0.5 °C were reported at lower temperatures. Reliable operation was achieved up to 380 °C. Canabal *et al.* [43] demonstrated wireless temperature sensing over a short range (1 cm) using frequency-multiplexed one-port resonators connected to a short loop antenna. They used the (0°, 144°, 24°) langasite orientation and operated near 200 MHz, with successful sensing reported at 900 °C.

## 5. SAW Gas Sensing—Sensing Layer Considerations

We now discuss recent work in our laboratory on SAW gas sensors. The sensing concept was illustrated in Figure 3(Bottom). A resistive gas-sensing layer covers part of the surface wave propagation path. The interaction between the surface wave and the resistive overlayer causes both attenuation and a change in velocity [44]. The effect of a resistive layer on the velocity has been calculated [45,46]; neglecting the mechanical effect of the sensing layer it can be shown that:

(1) $$\frac{\Delta \nu }{\nu_{0}}=\frac{K^{2}}{2}\frac{{\sigma_{s}}^{2}}{{\sigma_{s}}^{2}+{\left(\nu_{0}{ɛ}_{\text{eff}}\right)}^{2}}$$

where Δ*ν* is the change in velocity, *v*_0_ the velocity without a surface layer, *K*^2^ (≈0.0038 for the (0°, 138°, 27°) direction [47]) the electromechanical coupling coefficient, *σ_s_* the sheet conductivity of the sensing layer, and *ε_eff_* the effective dielectric permittivity.

**Figure 5.**
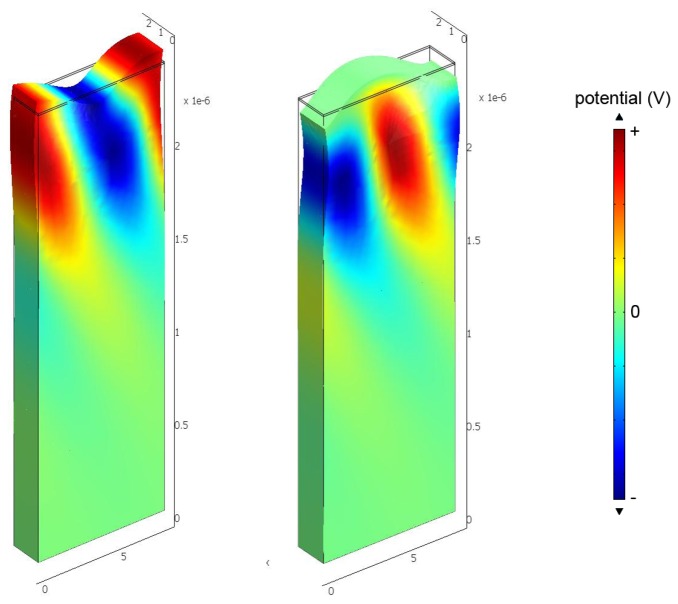
Simulated eigenmodes for 100 nm ZnO on langasite (**Left**, 348.5 MHz) insulating surface layer and (**Right**, 347.9 MHz) highly conductive surface layer. Color represents electric potential and the distorted shape is shown. A conductive surface layer shorts the electric field associated with charges induced on the surface.

**Figure 6.**
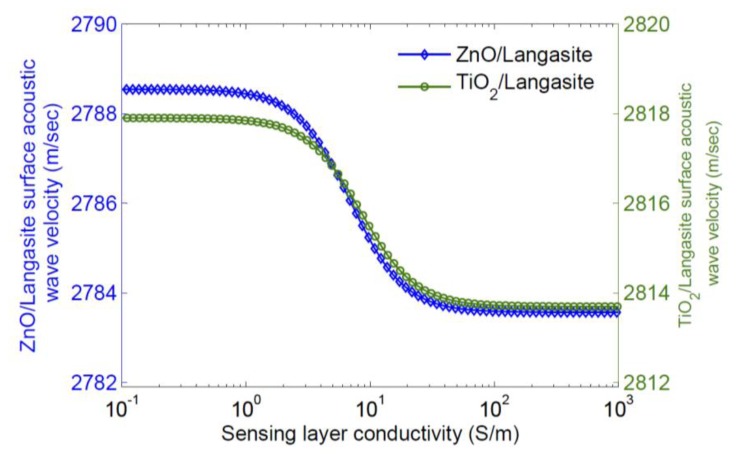
Dependence of velocity on film conductivity. Calculations are for a layer 100 nm thick.

Qualitatively, increasing the conductivity of the sensing layer tends to short the electric field associated with charges on the surface; this removes the effect of piezoelectric stiffening and results in a decrease in surface wave velocity. A significant dependence of velocity on sensing layer conductivity only occurs for a limited range in layer conductivity. Thus a sensing layer must be chosen so that it has the appropriate sheet conductivity range at the operating temperature. As most sensing layers have significant temperature dependence, it is necessary to simultaneously measure the sensor temperature for extraction of a gas concentration. The velocity change caused by a sensing layer can also be calculated using finite element simulation; this makes it possible to include all electromechanical effects of the sensing layer and also any buffer layer used [47]. Typical results are shown in Figures 5 and 6 [47], where the eigenmodes for langasite (0°, 138°, 27°) with 100 nm of ZnO have been simulated. Figure 5 shows the displacements normal to the surface for zero layer conductivity (Left) and high layer conductivity (Right). In these figures the color shows the electric potential; the effect of a high-conductivity sensing layer shorting the surface electric field is apparent. Figure 6 shows the simulated dependence of surface wave velocity on sensing layer conductivity. Very similar results are obtained for ZnO and TiO_2_, despite a large difference in sensing layer permittivity [47]. This is because the layers are thin enough compared to the wavelength that most of the energy stored in the electric field is stored above the sensing layer in air. We report measurements using an SnO_2_ sensing layer later. The SnO_2_ permittivity is 9*ε*_0_–14*ε*_0_ [48] and quite close to that of ZnO, and the velocity change is weakly dependent on the permittivity, additional simulations were not considered to be necessary.

We now consider the choice of sensing layer. Many metal oxides are oxygen defect conductors with an electrical conductivity that depends on the oxygen concentration [49,50]. As an example, we consider ZnO. Oxygen vacancies *V_ö_* can be reversibly formed by removal of oxygen atoms from an oxygen site $O_{o}^{x}$ according to:

(2) $$O_{o}^{x}↔V_{\ddot{o}}+2e^{\prime }+\frac{1}{2}O_{2}^{\left(g\right)}$$

where *e*′ represents a free electron and $O_{2}^{\left(g\right)}$ represents oxygen molecules in the gas phase. An equilibrium constant for this reaction given by:

(3) $$K=n^{2}[V_{\ddot{o}}]P_{o_{2}}^{1/2}$$

If doubly-ionized oxygen vacancies and electrons are the only charged species with significant concentration, charge neutrality requires that *n* = 2 [*V_ö_*] and we have:

(4) $$n^{3}=2{\text{KP}}_{o_{2}}^{-1/2}$$

Assuming that electrons are the dominant charge carrier, we find:

(5) $$\sigma =q\mu_{n}n\sim e^{-E_{A}/\text{kT}}\;P_{o_{2}}^{-1/6}$$

where the activation energy arises in part from the equilibrium constant and in part from temperature dependence of the electron mobility and the intrinsic free carrier concentration associated with promotion of electrons from the valence band to the conduction band.

**Figure 7.**
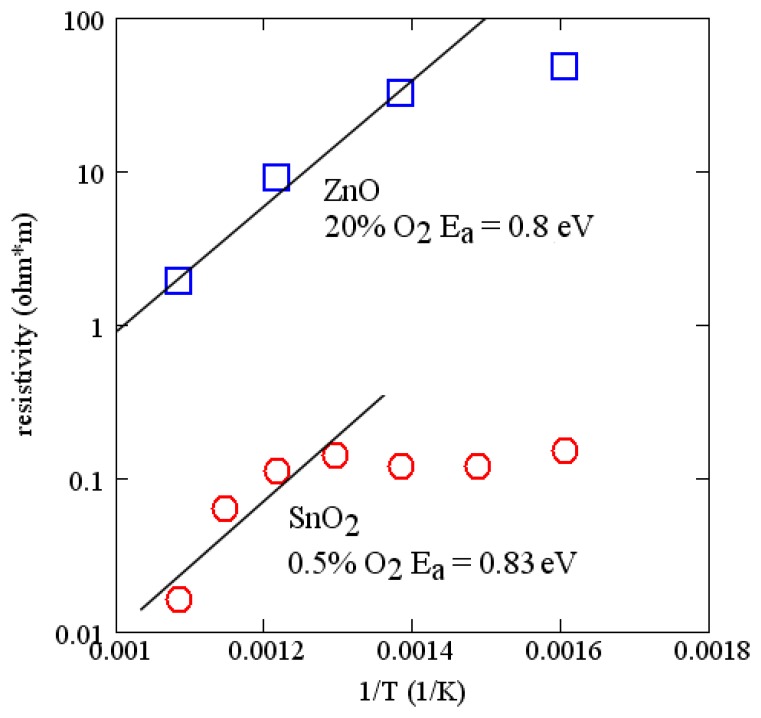
Measured resistivity for ZnO and SnO_2_ as a function of reciprocal temperature at constant oxygen concentration.

Activation energies for many of the common oxides are of the order of 1 eV. The measured conductivity of ZnO and SnO_2_ as a function of temperature are shown in Figure 7. These measurements were made on 100 nm layers deposited by RF reactive sputtering in argon and oxygen. Sputter conditions are reported in Table 1. Comparing with Figure 6, useful changes in velocity can be expected for temperatures of the order of 700 °C. The measurements on SnO_2_ support a similar activation energy (∼0.8 eV) and are consistent with a report in the literature [51]. SnO_2_ has lower resistivity and is suited for sensing at lower temperatures. Because of the relatively strong temperature dependence of these simple oxides, the temperature range of operation of a sensor is limited, although it can be extended somewhat by using two sensing layers on the same device. Alternatively, there are more complex oxides that have much lower temperature dependence [52,53].

**Figure 8.**
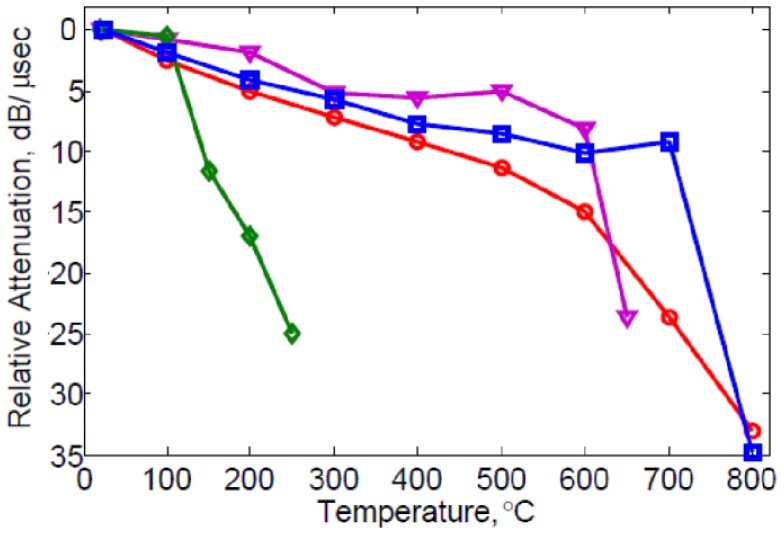
Surface acoustic wave attenuation of (0°, 138°, 27°) langasite SAW devices with different overlayers as a function of temperature. Measurements were performed at 335 MHz. Blue square 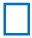: non-layered langasite; purple triangle 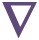: sputter-coated 200 nm ZnO/langasite; red round 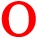: spin coated 200 nm SiO_2_/langasite; green diamond 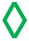: spin coated ZnO (140 nm;/SiO_2_ (200 nm)/langasite.

Another important aspect of the sensing layer is its effect on the surface wave attentuation. This attenuation is not only associated with *electrical* loss within the film. It is well known that the electrical loss in the film peaks at the conductivity that corresponds to the largest slope in the Δ*v-σ* curve [45,46], and that it approaches zero for large and small conductivity. There is additional loss observed either with insulating [47,54] or conducting overlayers [33,34] (that is, layers with conductivity high enough or low enough that electrical dissipative losses are insignificant). Figure 8 compares the relative attenuation for different overlayer materials. Spin-coated ZnO causes considerable attenuation even at temperatures where electrical conductivity is negligible. Increasing losses at higher temperature due to metal overlayers have also been reported [33,34]. Probably this attenuation is a consequence of internal mechanical losses and can be expected to be dependent on the composition and structure of the material. When layers are rough or nonuniform additional losses due to scattering of the surface waves can be anticipated. Losses are a concern not only for sensing layers but also for buffer and passivation layers.

## 6. SAW Sensor Design and Fabrication

**Figure 9.**
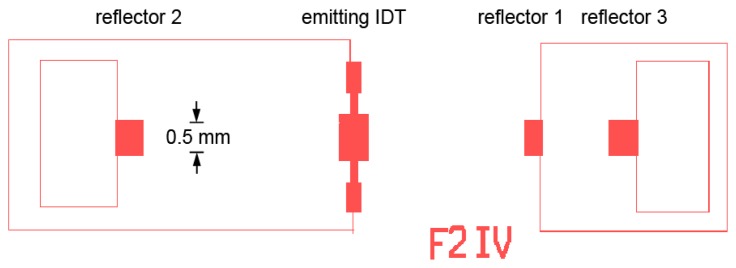
Mask layout for SAW sensor used in this work.

We now consider the design and fabrication of SAW gas sensors. Figure 9 shows the mask layouts for a SAW design that has been used in our earlier experiments [55]. Mask parameters for SAW devices used in the gas sensing experiments to be discussed later are shown in Table 2.

In designing SAW sensors, several aspects must be considered. In general it is desirable to have large IDT apertures to minimize the effect of diffraction and to maximize signal strength. However the metallizations typically used for high-temperature sensing have relatively high resistivity (Pt has resistivity about four times greater than aluminum). The practical thickness of these metals is limited by cost and also difficulty in performing lift-off patterning with thick layers. As a result, devices with large aperture will have high finger resistance, resulting in excess loss [47]. An aperture of 50*λ* was chosen based on this consideration.

The spacing between emitting and reflecting IDTs involves a tradeoff. With large spacing the change in round-trip time is maximized; however increasing the spacing also increases the attenuation associated with the langasite itself, mechanical losses in the overlayer, and resistive losses in the sensing layer. As a result, moderate spacing (Figure 9) is preferred.

We have used multiple reflectors, typically three in total, on both sides of the emitting IDT. Langasite is known to exhibit NSPUDT behavior [56], which suggests that placing reflectors on only one side would be preferable. However if the unidirectional behavior is modeled using the *P* matrix it can be shown that improved emission in one direction does not result in an overall improvement in signal for reflectors on one side when reflection and detection are considered [57]. Experimentally, we find that all reflections are observed with similar intensity.

We have previously reported initial results of experiments on oxygen gas concentration measurements [58] and simultaneous gas concentration and temperature measurements [59] using ZnO sensing layers. It should be noted that ZnO is itself a piezoelectric material; however much larger thicknesses than used in our work as a sensing layer are needed to significantly alter the surface wave propagation [60]. A subsequent paper provided additional details of the experiments [47]. Those results convincingly showed that oxygen detection had been achieved and also showed that conductivity changes in the sensing layer were the reason for the oxygen response. It was necessary to rule out the possible influence of conductivity changes in the langasite substrate itself, as oxygen defect conductivity also occurs in langasite [29]. The conductivity of langasite is only measurably dependent on oxygen concentration for considerably lower partial pressures of oxygen than studied in our experiments, and in addition the absence of an oxygen response by SAW temperature sensors provided further confirmation that the langasite conductivity is not a factor.

Despite a clear demonstration of oxygen sensing, the reported results showed some practical problems. Aging of the sensing response was observed. Also, the response time was quite long, leading to incomplete saturation of the measured phase change. In this paper, we report studies directed at understanding and mitigating the drift in oxygen response. These studies were largely conducted using SnO_2_ sensing layers.

## 7. Gas Sensing Experiments with SnO_2_ Sensing Layers

**Figure 10.**
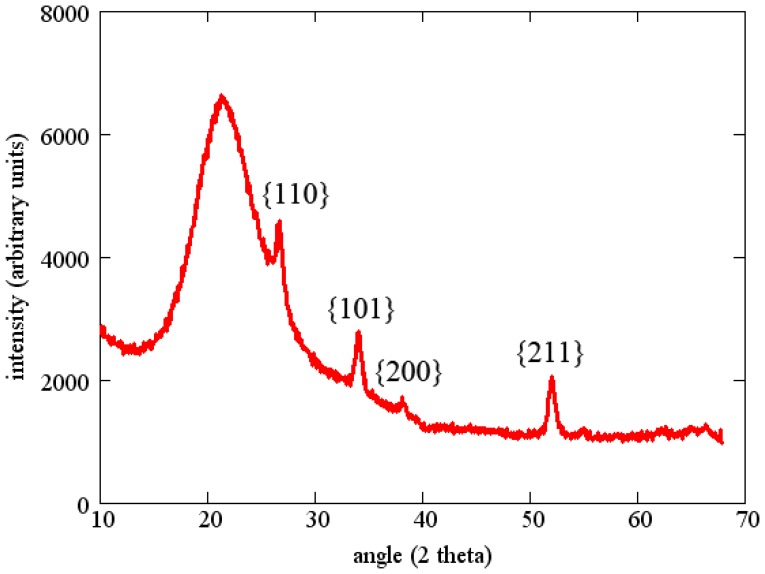
X-ray diffraction scan of SnO_2_ layer after annealling.

Tin oxide sensing layers were deposited by reactive sputtering of a pure tin target in a 50% Ar/50% O_2_ gas mixture. The total pressure during sputtering was 4 mTorr and the RF power was 100 W to a 75 mm diameter sputter gun. The thickness of the SnO_2_ layer in the following experiments was about 100 nm. All sensing layers were annealed for one hour at 700 °C in air before starting measurements. This anneal was intended to stabilize the grain size and also to fully oxidize the film. The resulting films were polycrystalline without a predominant texture as determined by X-ray diffraction (Figure 10).

**Figure 11.**
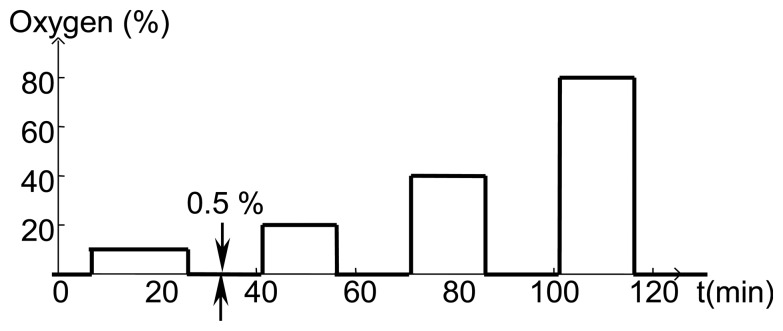
Gas flow sequence used for measurements presented in this paper. Oxygen concentration steps were 10%, 20%, 40% and 80% above the 0.5% baseline.

Before fabricating SAW devices, 100 nm layers of SnO_2_ were deposited on fused silica substrates and the resistivity was measured using the van der Pauw technique as a function of temperature and oxygen concentration. All gas sensing studies were performed using the same gas flow sequence (Figure 11) where the oxygen concentration was varied and the balance of the gas flow was nitrogen. At the start of experiments, the furnace tube was filled with room air and as a result the first gas concentration step sometimes showed anomalous behavior. The total gas flow was kept constant in order to prevent any temperature transients associated with changing flow rate.

**Figure 12.**
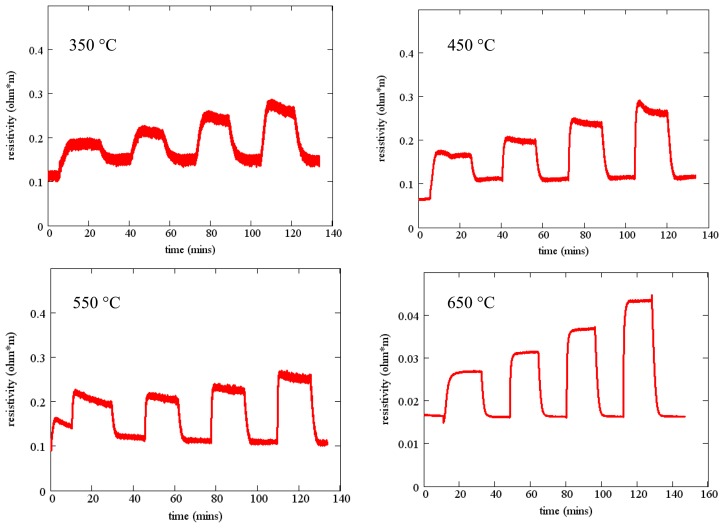
Resistivity as a function of time for SnO_2_ deposited on fused silica. Each plot shows the resistivity as a function of time when the oxygen concentration is varied according to Figure 11.

Measured resistivity as a function of time for the gas flow sequence of Figure 11 is shown in Figure 12 for four temperatures. These measurements show minimal baseline drift and oxygen response that increases monotonically with oxygen concentration. In addition, the time constant of the gas response is short, especially at higher temperatures. The resistivity at constant oxygen concentration decreases with temperature, yielding an activation energy of about 0.8 eV (Figure 7).

We now consider the oxygen response of a SAW device with the same SnO_2_ sensing layer. Measurements were made using the interrogation electronics described in detail elsewhere [61]. Briefly, the interrogation electronics produce an exciting pulse which is a windowed sinusoid at the operating frequency of the SAW device (∼327 MHz in these experiments). This exciting waveform is applied to the emitting IDT of the SAW device. A surface acoustic wave is generated which propagates beneath the sensing layer and is reflected by the reflecting IDT. When this reflected pulse returns to the emitting IDT it is converted into an electrical signal. The interrogation electronics performs phase detection and averaging. Phase is computed relative to a reference signal that can be either an attenuated version of the exciting waveform or a reflection from a different IDT. The result is a plot of detected phase difference as a function of time. The phase change is directly proportional to the change in surface wave propagation velocity.

**Figure 13.**
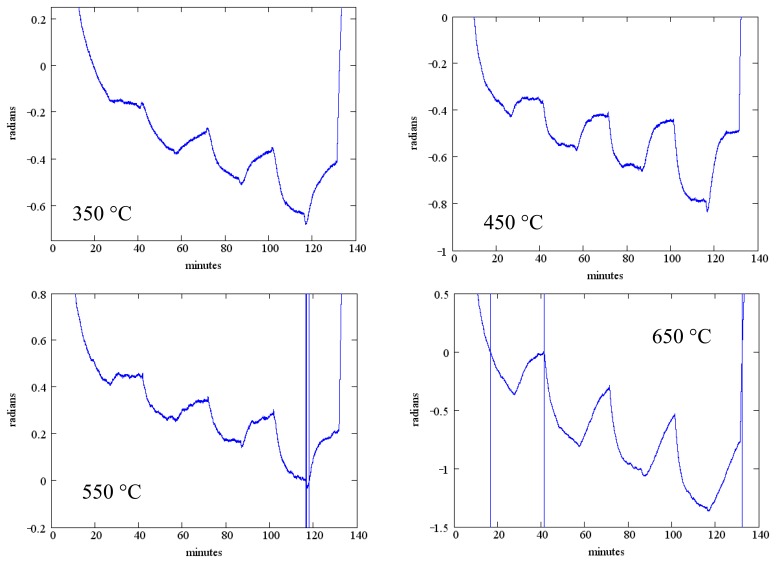
Measured phase change as a function of time for SAW device with 100 nm SnO_2_ sensing layer. Each plot shows the phase as a function of time when the oxygen concentration is varied according to Figure 11.

The measured phase change as a function of temperature is shown in Figure 13 for SAW device F2 (Table 2). The phase reference in these measurements is the exciting waveform and the data presented is for the reflection from the IDT located closest to the emitting IDT. Data presented is for the same temperatures as Figure 12 and the same gas flow sequence; as before, an anneal at 700 °C for one hour preceded these measurements. These measurements were performed in the wired mode.

While the measured phase shift shows a clear response to the oxygen concentration, there is considerable baseline drift and the oxygen response does not reach saturation within the 20 min dwell time.

**Figure 14.**
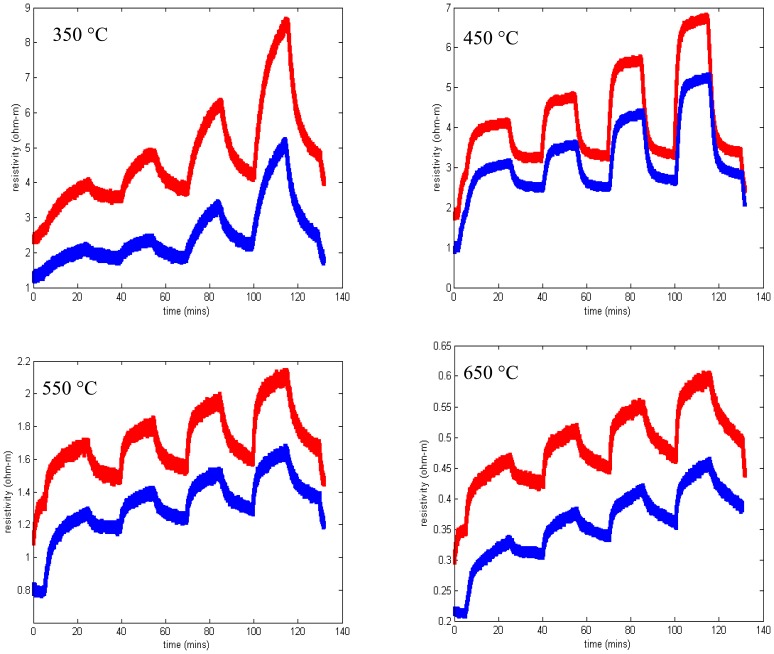
Resistivity measurements of 100 nm SnO_2_ on langasite substrate from 350 °C to 650 °C. The blue trace is the first set of temperature measurements and red is the second set of measurements. Each plot shows the resistivity as a function of time when the oxygen concentration is varied according to Figure 11.

It can be clearly demonstrated that this degradation in sensing response is associated with some interaction between the SnO_2_ sensing layer and the langasite substrate. We repeated measurements of the sensing layer resistivity as a function of oxygen concentration for an SnO_2_ film on a langasite substrate (no SAW device structure). The sequence of measurements from 650 to 350 °C was repeated twice in order to check reproducibility. These results are presented in Figure 14. Similar to the SAW sensor measurements, these measurements show pronounced drift and poor saturation of the gas response. In addition, comparison of the two sets of measurements shows a consistent shift to higher resistivity in the second set of measurements, although the fractional resistivity change caused by the gas ambient is relatively stable.

Possible explanations for this behavior are either (1) interdiffusion between the langasite and the sensing layer, altering the defect chemistry of the sensing layer, and hence the oxygen response; or (2) oxygen diffusion into the langasite from the sensing layer. We consider these two possibilities in turn.

**Figure 15.**
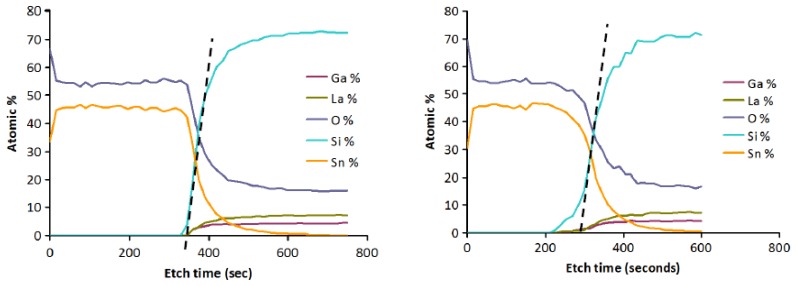
XPS depth profiles (**Left**) as-deposited and (**Right**) after 700 °C one hour anneal, and a sequence of gas sensing measurements.

Evidence for interdiffusion between the langasite substrate and the sensing layer may be obtained by comparing atomic depth profiles before and after annealing. XPS depth profiles of the as-deposited and annealed SnO_2_/langasite materials are shown in Figure 15. The transition region (the area near the dashed line) appears to be not as sharp after annealing, consistent with a small amount of interdiffusion between the two layers. Note that a small amount of an ionic species could significantly influence the oxygen vacancy concentration because the oxygen vacancy concentration is small at these temperatures.

**Figure 16.**
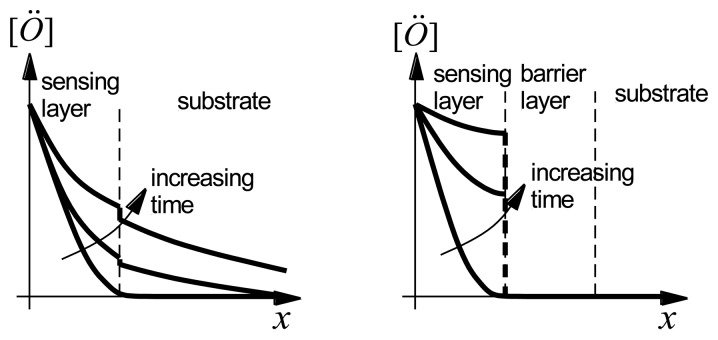
Oxygen profiles (**Left**) without barrier layer and (**Right**) with barrier layer.

Alternatively, diffusion of oxygen vacancies into the langasite substrate could account for the observations. Figure 16(Left) shows the qualitative behavior of the oxygen vacancy concentration after an increase in the oxygen partial pressure at the surface of the sensing layer. The oxygen vacancy concentration increases with time in both the sensing layer and in the langasite substrate. Because the substrate acts as a sink for oxygen vacancies, a steady state oxygen vacancy concentration in the sensing layer is approached very slowly. Diffusion of oxygen vacancies can account for the long time constants and baseline drift shown in Figures 13 and 14.

In either case, we can expect that a barrier layer would have a beneficial effect as it would prevent either interdiffusion or oxygen vacancy diffusion into the substrate (Figure 16(Right)). We discuss the experimental results obtained with barrier layers in the following section.

## 8. Effect of Barrier Layers for Gas Sensing

**Figure 17.**
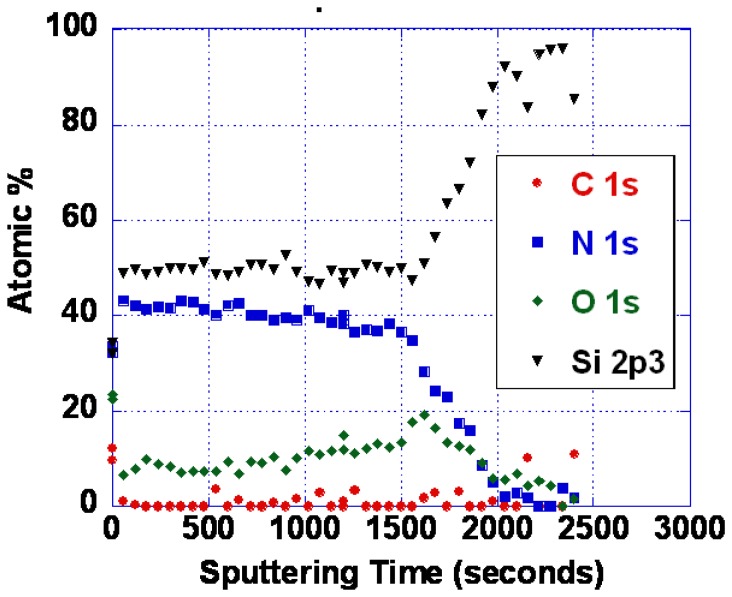
X-ray photoelectron spectroscopy depth profile of silicon oxynitride layer deposited on a Si substrate.

**Figure 18.**
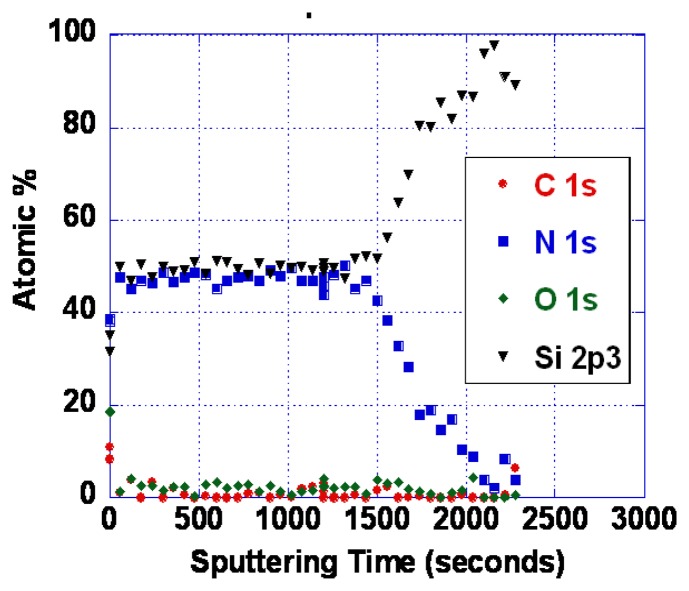
X-ray photoelectron spectroscopy depth profile of silicon oxynitride layer deposited on a Si substrate.

As noted earlier, the problems with poor response time and baseline drift could be caused by either oxygen vacancy diffusion or diffusion of metal ions. As it is not yet known which diffusing species is responsible, we have so far only explored SiO*_x_*N*_y_* barrier layers. Silicon nitride is known to be an excellent barrier for oxygen and water vapor [62]. Diffusion of oxidizing species through silicon dioxide is slow below about 800 °C, as evidenced by the very low rates of thermal oxidation [62]. We have performed some experiments using various compositions of SiO*_x_*N*_y_*. Barrier layers of 100 nm thickness were deposited by reactive sputtering in an Ar/N_2_ mixture. Even with no oxygen deliberately introduced, some oxygen is typically present in the film. This is because there is inevitably trace water vapor present, and the N_2_ bond strength is high making oxygen incorporation significant even when the water vapor partial pressure is small. Figure 17 shows an XPS depth profile of an oxynitride layer resulting from deposition in 25% N_2_/75% Ar. With more complete purging and pumping of the system, nearly pure silicon nitride was also deposited (Figure 18).

**Figure 19.**
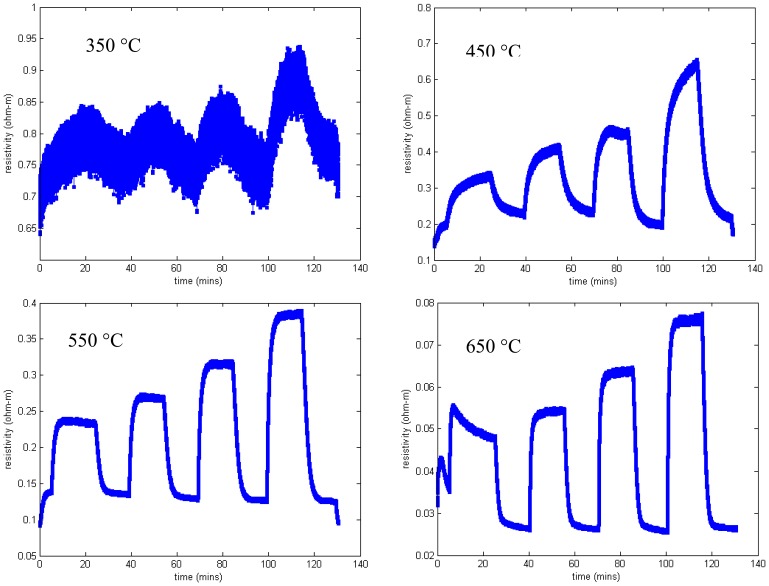
Resistivity measurements of SnO_2_ on oxynitride on langasite as a function of time. Each plot shows the resistivity as a function of time when the oxygen concentration is varied according to Figure 11.

We observed that silicon nitride and silicon oxynitride layers exhibited poor adhesion to langasite and fused silica substrates. This is not surprising as silicon nitride deposited by other techniques is well known to exhibit a high degree of stress that ranges from compressive to tensile depending on deposition conditions. Tensile-strained silicon nitride is particularly susceptible to peeling and delamination [63]. Even so, we were able to complete some resistivity measurements for SnO_2_ with a silicon oxynitride buffer layer (profile shown in Figure 17) on a langasite substrate. These measurements are shown in Figure 19, whereas before the measurements were preceded by an anneal for one hour at 700 °C followed by resistivity measurements beginning at 650 °C and continuing at lower temperatures.

Resistivity measurements at 650 °C and 550 °C show generally good response time and little to zero drift, similar to the results obtained on fused silica substrates (Figure 12). The data at 450 °C shows longer time constants and incomplete saturation and at 350 °C the measurements were unstable. At the end of the experiment some localized areas of delamination were apparent. It is possible that degraded contacts contributed to the unstable measurements at 350 °C.

**Figure 20.**
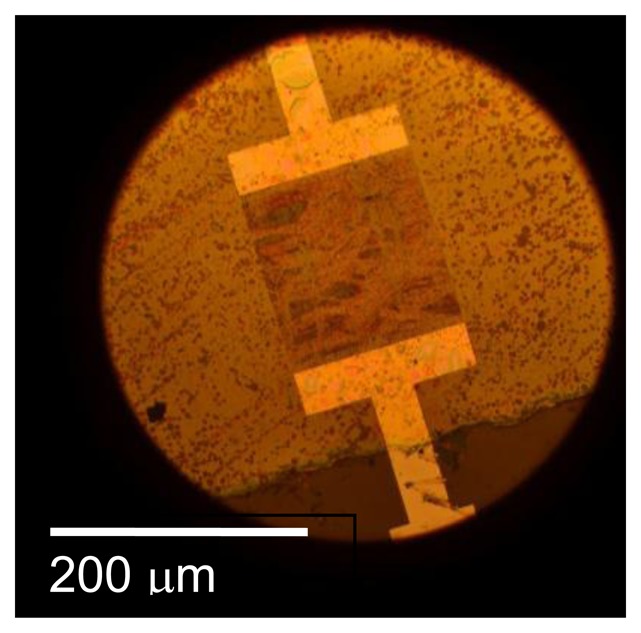
SAW device with SnO_2_ and silicon oxynitride barrier layer after annealing at 700 °C.

These measurements support the hypothesis that a barrier layer would considerably improve the stability and response time of SAW gas sensors. Unfortunately it was not possible to test this hypothesis with the oxynitride buffer layers. Two attempts were made with oxynitride buffer layers with different oxygen concentration and in both cases delamination was observed after annealing at 700 °C (Figure 20). No reflections were observed for this device even at room temperature. This is not unexpected as scattering and attenuation of surface acoustic waves by this highly nonuniform layer is expected to be strong.

Some further confirmation of the effectiveness of the barrier layer was obtained from other experiments with ZnO sensing layers (not reported here). Briefly, improved response time and reduced drift was observed in a SAW sensor when a silicon oxide buffer layer was used.

Finally, we can show that the measured SAW gas response without a barrier layer is consistent with the measured sensing layer resistivity. Using Equation (1) above, we can predict the phase change resulting from a given sensing layer resistivity change. We use the measured resistivity from the van der Pauw method for an SnO_2_ layer deposited on a langasite substrate. The electromechanical coupling coefficient in expression (1) is well-established, leaving only the effective permittivity as an adjustable parameter. The phase is related to the velocity change Δ*ν* through:

(6) $$\theta =2\pi f_{0}t_{0}\cdot \frac{\Delta \nu }{\nu_{0}}+\theta_{0}$$

where *f*_0_ is the SAW center frequency, *t*_0_ the round trip delay time, *ν*_0_ the surface wave velocity with no sensing layer, and *θ*_0_ a constant.

**Figure 21.**
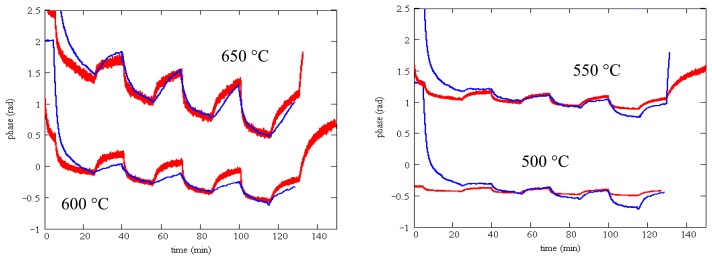
Measured phase changes for SAW device with SnO_2_ sensing layer (blue) and calculated phase change from measured resistivity (red).

Figure 21 shows the predicted phase change from the resistivity measurements (red) and the measured phase change (blue). In these plots the constant θ_0_ in the predicted phase change has been adjusted to match the two sets of data. The curves are for a single value of the effective permittivity (*ε* = 10) and using *K*^2^ = 0.0038 [47]. The agreement between predicted and measured phase change is satisfactory apart from an initial transient that varies depending on the gas composition in the furnace when the experiment was started. Predicting the phase change using measured resistivity on fused silica (rather than the measured resistivity on a langasite substrate) results in considerably worse agreement.

We have therefore shown that the zero drift and long time constants observed in earlier experiments are not an intrinsic characteristic of SAW gas sensors. Based on these results, we are confident that sensors fabricated with an appropriate barrier layer will mitigate the problems of zero drift and long time constants. A major consideration will be the adhesion of the barrier layer when exposed to elevated temperatures. Possible barrier layers include silicon nitride with well-controlled built-in stress, silicon oxide, and aluminum oxide. If these barrier layers cover the interdigitated transducers then a further benefit will be protection of the electrodes from degradation at high temperatures.

## 9. Prospects for Wireless Harsh-Environment Gas Sensors

To date we have tested gas sensors only in wired mode and up to temperatures of about 700 °C. In general the maximum temperature of operation for langasite SAW sensors appears to be limited by attenuation in the langasite substrate and any other materials on the acoustic path. In a gas sensor, attenuation is caused by mechanical losses in the sensing and buffer layers and by resistive losses in the sensing layer (when the sensing layer is rough or discontinuous there will be additional attenuation). Wireless sensing incurs additional losses in the RF link so this is a particularly serious issue for gas sensing. The additional losses are not present when the SAW device is used as a temperature sensor; temperature sensing has been achieved up to 900 °C even in wireless mode. Operation of gas sensors in wireless mode at high temperatures will require reductions of the acoustic losses and/or improvements in SAW design. It is possible that different sensing layers or a different substrate material will have lower acoustic losses.

Wireless mode operation also requires fabrication of an antenna compatible with high temperature operation. Ideally that antenna should be compact and well-matched to the SAW device. In prior work inductive matching has been used for high-temperature wireless sensing [41] at 415 MHz. At a higher frequency of 2.45 GHz a compact slot antenna has been reported [42] although attentuation in the SAW device was a problem even at 300 °C. We have reported a meander-dipole design that can be designed to have a conjugate match with the SAW IDT impedance without the need for matching components [64,65]. A conjugate match is achieved when the the antenna is slightly longer than at resonance so that the imaginary part of its impedance is equal to the imaginary part of the impedance the emitting IDT. The real part of the impedance depends on the number of finger pairs and width of the emitting IDT [47] and is close to the real part of the impedance of the meander dipole for our IDT design.

**Figure 22.**
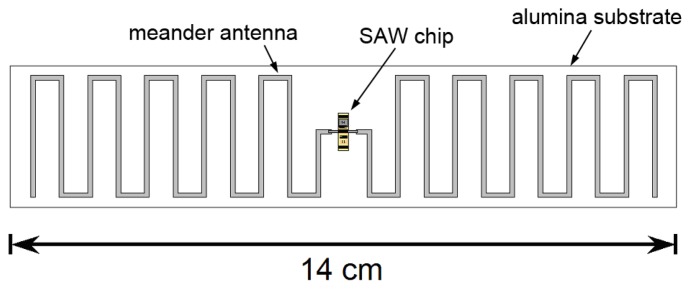
Conceptual design of SAW gas sensor.

Such an antenna could be fabricated on a ceramic substrate using screen-printed conductive ceramic paste to form the antenna conductors and connections to the SAW device. Figure 22 shows a conceptual design for a wireless sensor including an antenna, designed for operation at modestly higher frequencies near 433 MHz. We see no fundamental barriers to operating a wireless gas sensor of this type up to at least 700 °C, with possible extension to higher temperature operation with improved materials with less intrinsic loss.

## 10. Conclusions/Outlook

We have reported recent work on the development of SAW gas sensors for harsh environment applications. SAW sensors have been fabricated and characterized using tin oxide sensing layers on langasite substrates. Sensors with the tin oxide layer directly on the langasite substrate have degraded transient response and pronounced drift. By employing a suitable barrier layer between the sensing layer and the SAW device we expect significantly improved gas sensing characteristics, although further development of a barrier layer that adheres well during high-temperature exposure will be necessary.

## Figures and Tables

**Table 1.** Sputtering conditions for ZnO and SnO_2_ layers.

**Material**	**Target**	**Thickness**	**Gas Mixture**	**Power**	**Duration**
ZnO	25 mm Zn	100 nm	1 mT O_2_, 3 mT Ar	50 W	30 min (Figure 7)
ZnO	25 mm Zn	100 nm	2 mT O_2_, 2 mT Ar	50 W	6 min 24 s (Figure 8)
SnO_2_	75 mm Sn	100 nm	2 mT O_2_, 2 mT Ar	100 W	12 min 20 s

**Table 2.** Mask parameters for gas sensor experiments.

**Mask ID/ aperture**	**Electrode Pt Thickness**	**Transmitter (pairs)**	**Reflectors (distance/pairs)**
F2/50*λ*	100 nm	50	3.2 mm/50 (left) 2.56 mm/30, 3.85 mm/50 (right)
F10/50*λ*	100 nm	50	3.2 mm/50 (left) 2.56 mm/10. 3.84 mm/50 (right)
